# University students’ understanding of contract cheating: a qualitative case study in Kuwait

**DOI:** 10.1186/s40468-022-00208-y

**Published:** 2022-12-02

**Authors:** Inan Deniz Erguvan

**Affiliations:** Gulf University for Science and Technology, West Mishref, Kuwait

**Keywords:** Contract cheating, Online learning, Academic integrity, Student perspectives, Focus group

## Abstract

Contract cheating, or students outsourcing their assignments to be completed by others, has emerged as a significant threat to academic integrity in higher education institutions around the world. During the COVID-19, when traditional face-to-face instruction became unsustainable, the number of contract cheating students increased dramatically. Through focus group interviews, this study sought the perspectives of 25 students enrolled in first year writing in a private higher education institution in Kuwait during the pandemic in 2020–2021, on their attitudes towards contract cheating. MAXQDA 2020 was used to examine the data. The participants believe that the primary motivations for engaging in contract cheating are mainly the opportunities presented by online learning and the psychological and physical challenges they experienced during online learning. Those who did not cheat had some shared traits, such as a competitive spirit, confidence in their talents, and a strong desire to learn. Additionally, those with high moral values avoided cheating. To combat contract cheating, students believe that teaching and evaluation techniques should be drastically altered and that students should be educated about plagiarism, while institutions should impose tougher sanctions on repeat offenders.

## Introduction

Universities met their teaching commitments through remote teaching because traditional face-to-face teaching temporarily became unsustainable due to the COVID-19 pandemic that hit the world between 2020 and 2022. Because students were not physically present in the classroom during this time, many higher education institutions conducted tests through digital platforms or replaced exams with essays and other types of written tasks (Gamage et al., 2020). The technology and infrastructure required to join online sessions, concerns about privacy as information technology devices demanded access to students' cameras, microphones, and desktop, and, most importantly, questions about academic integrity have been raised as a result of alternate methods to assessment. Replacement of exams with writing and/or take-home assignments constituted a danger to academic integrity, necessitating the adoption of fraud-free methods (Almeida & Monteiro, 2021).

Academic dishonesty, which refers to committing or contributing to dishonest acts in teaching, learning, research, and related academic activities (Cizek, 2003; Whitley & Keith-Spiegel, 2001), has long been a source of concern in higher education and has been on the rise in recent years. According to some estimates, up to 75% of university students have engaged in some type of academic misconduct during their academic career (Brimble & Stevenson-Clarke, 2005; McCabe & Bowers, 1994).

Plagiarism is one of the most persistent issues confronting higher education institutions, and it can take many forms, including “copy and paste” without citing the source; patch-writing; providing incorrect or incomplete citations or references; presenting or citing a secondary source as a primary source; ghost-writing; and contract cheating (De Jager & Brown, 2010; Ellery, 2008; Ellis et al., 2018; Park, 2003; Zafarghandi et al., 2012). Clarke and Lancaster (2006) coined the term “contract cheating” to characterize the unacknowledged usage of materials prepared by another person or entity involved in the sale of academic resources. Students outsource their coursework to others to do, usually for a fee, and then present it as their own. Contract cheating, according to many people, is a growing problem that most academic institutions are dealing with. To make matters worse, it is difficult to spot ghostwritten work because it is a new piece of writing tailored to course requirements and specific assignments (Ali & AlHassan, 2021).

Although contract cheating is common in both traditional face-to-face and online settings, it is more likely to take place in the latter. There are some strong indications that contract cheating went rampant during the pandemic and became a significant COVID-19 side effect for higher education institutions. According to Lancaster and Cotarlan (2021), the number of requests for answers to academic questions on a popular student website jumped by over 200% during the pandemic. Lancaster (2020) found that a website providing essay writing services expanded the number of tutors they recruited and was able to offer discounts due to the increased profitability. Similarly, during the first COVID-interrupted semester, additional research found that university students believed their colleagues cheated when classes went online (Daniels et al., 2021) and that their willingness and pressure to cheat were stronger online than in-person (Walsh et al., 2021). Likewise, King and Case (2014) discovered that throughout a 5-year period, the number of students who self-reported academic cheating increased, and around 75% of students said it was easier to cheat in online assessment.

There are several possible explanations for why students engaged in contract cheating in online education more often than in person education. Psychological distance adversely affected interpersonal relationships; and the internet obscured the line between academically honest and dishonest behavior (Eshet, 2022). Sudden campus closures and abrupt transition to online teaching modals provided more opportunities for students to complete assignments with online assistance. Furthermore, essay mills saw the lack of face-to-face interaction and proctoring on campus as an opportunity and used aggressive marketing methods to attract students. Through social media, students quickly became aware of the possibilities of a wide variety of options to carry out contract cheating; as a result, contract cheating has emerged as a real threat to academic integrity (Erguvan, 2021; Hill et al., 2021, Bautista & Pentang, 2022).

## Review of literature

Academic dishonesty is a complicated system including a variety of components that interact in unanticipated ways. Due to the vast number of paper mills, full-text databases, and collaborative web pages, many researchers attribute the rise in academic dishonesty to the increased usage of the internet, which generates “opportunities” for cheating (Peytcheva-Forsyth, et al., 2018; Townley & Parsell, 2004). Students engage in academic dishonesty for a variety of reasons, according to researchers: desire for high grades, procrastination, time pressure to complete assignments or study for tests, lack of organizational skills, fear of failing a course (loss of time and money), lack of understanding of academic dishonesty, and plagiarism not being considered a serious offense (Eshet et al., 2012; Jone, 2011; McGee, 2013). Academic dishonesty is influenced by social factors including peer pressure, social attitudes and norms about academic dishonesty, or domestic job market circumstances (Carpenter, et al., 2006; Gallant & Drinan, 2006). A “competitive culture” to earn excellent grades or succeed in school (Roberts & Hai-Jew, 2009). Furthermore, if the dominant culture does not regard academic dishonesty as a significant problem that requires attention, such situations will be handled on an individual basis, and formal consequences will rarely be pursued. The existence of an institutional policy on academic integrity, code of honor, and effective disciplinary procedures performed by educational institutions are all institutional elements that may affect academic dishonesty (Roberts & Hai-Jew, 2009; Vilchez & Thirunarayanan, 2011).

Many theories have been developed to describe why and how students engage in plagiarism and what factors play a role in this. Plagiarism has been commonly understood using theoretical frameworks originating from criminology literature which conceptualizes students as delinquents; however, that is rather problematic and ineffective in the long run (DiPietro, 2010). Some other theories could be listed as deterrence theory, rational choice theory, neutralization theory, planned behavior theory, situational ethics, social learning theory, self-control, and rational choice theory (DiPietro, 2010; Sattler, et al., 2013). This research was guided by social learning theory and rational choice theory.

Social learning theory by Albert Bandura could be applied to explain the learners’ plagiarism behavior. Bandura (1997) theorizes that learning is a cognitive-process that takes place in a social-context and can occur through “the influence of example” by observing a behavior and/or the consequences of the behavior. Therefore, if learners discover their fellow classmates plagiarizing and getting high grades and receiving nominal or no punishment at all for these acts, they will also feel inclined to adopt cheating. If a behavior is learned with a perceived negative consequence associated with it, then an individual is more likely to inhibit that behavior for him- or herself. However, positive reinforcement, which can also mean not having a negative consequence associated with the behavior, may encourage behaviors, whether they are positive or negative. (Denler et al., 2014).

Students choose to plagiarize in their assignments or tests for a range of reasons, and it is possible to examine the students’ motivation within the framework of rational choice theory, according to which every individual follows the principle of maximum utilization when they have to make a decision (Hawes, 2020). Individuals compare potential advantages to possible costs entailed by their decision and the course of action is chosen after weighing the advantages and disadvantages of all possible alternatives. Therefore, the decision to cheat and plagiarize results from a cost–benefit analysis. On the one hand, plagiarism offers some benefits: allowing students to finish the work quickly and save time and improve their grades; on the other hand, there are some counter-factors such as the risk of being caught. In case of plagiarism, potential losses would become real if the fact of plagiarism were to be discovered, which is not always likely. The consequences of plagiarism for students might include unsatisfactory marks for the assignment, reproach by the teachers, or other disciplinary punishments. Nevertheless, such measures do not seem to be significant enough to have the possibility to prevent students from plagiarizing. The risk of being caught has a medium negative effect on cheating, the fear of punishment has a small negative effect, and the importance of the outcome has a medium positive effect (Whitley, 1998).

Even though large numbers of students are claimed to partake in contract cheating in Kuwaiti higher education, such as purchasing papers from shops on campus that seemingly provide only printing and photocopying services (Al Jiyyar, 2017), there is little research on contract cheating in Kuwait. Indeed, a literature review by Ahsan et al. (2021) identifies research deficits outside of Australia, the UK, and Canada, as well as in contexts of contract cheating such as society, culture, and religion. Contract cheating during and after COVID-19 is another dimension that has received insufficient attention.

As a result, the purpose of this study is to investigate students' opinions of contract cheating occurring in first year writing classes in a private university context in Kuwait, a country that has had little research done in this area. The questions that will guide the study are as follows:

1. Why did more students engage in contract cheating during the pandemic?
2. What stopped students from engaging in contract cheating?
3. What consequences did contract cheating students face, if any?
4. What should be done to curb contract cheating?

## Methods

### Design

In this exploratory case study, participants’ perspectives were acquired through focus group interviews, which is a popular strategy for acquiring qualitative data (Morgan, 1996). The strength of focus groups is that they allow participants to interact in groups which can provide insights into the causes of complicated actions as a result of group interaction (Carey & Smith, 1994; Morgan & Krueger, 1993). Because members simultaneously question and explain themselves to one another, focus groups are more than the sum of separate individual interviews. Because of the sensitive nature of the subject, the researcher determined that a group discussion, rather than individual interviews, would yield more insightful data.

### Participants

Regarding the number and size of focus groups, different authors have varied advice and references. Various researchers have noted that sizes of focus groups may range from four to five, six to eight, and even eight to twelve people, and some have even suggested that there are no universal standards for the best number of focus groups (as cited in Gundumogula, 2020).

The researcher recruited twenty-five students for this study and five focus group interviews were scheduled, with each interview containing five to six students. There were eleven females and fourteen males among the participants. Purposeful sampling was used as the sampling method. Other faculty members in the department were contacted and asked to provide a list of potential participants. The list of participants suggested by faculty members was screened for eligibility to see if they met the following inclusion criteria:

- Fluent in English.
- Currently registered in a course during the 2021–2022 Fall term in the university.
- Enrolled in a university and attended online classes in the previous academic year, 2020–2021.

Diversity in gender, discipline, and academic performance was observed in recruitment. The potential participants who were selected were contacted by e-mail. Official invitations to the online meeting were sent via emails to those expressed interest in attending.

The informed consent form which was approved by the Institutional Review Board included a detailed description of the interview process and confidentiality information. The participants were asked to read and sign these forms before the interview took place.

### Researcher’s role

In qualitative research, because the researcher is the instrument in semi structured or unstructured qualitative interviews, unique researcher attributes have the potential to influence the collection of empirical materials (Pezalla et al., 2012); therefore, explicitly identifying oneself is more important than it is in quantitative research. In this study, the researcher is a faculty member in the same institution where the research took place, thus, familiar with the plagiarism and contract cheating habits and attitudes of Kuwaiti undergraduate students. She also included some of her own as well as her colleagues’ students in the study. She stated clearly at the beginning of the study that student responses will not be used for any course related assessment and evaluation and the collected data will be limited to research only. She only joined the focus group meetings as a listener, and another trained colleague moderated these meetings. Although every effort has been made to ensure objectivity, certain biases may remain, and these biases may shape the way the data is collected and the participants’ experiences are interpreted in this study.

### Data collection

All sessions were conducted online, due to the pandemic restrictions still in place, and in English, between November and December 2021. The sessions were recorded and simultaneously transcribed using the built-in function of the online meeting platform. The participants were informed of recording at the time of recruitment, as well as the beginning of each session.

Each online focus group lasted between 60 and 90 min that allowed in depth discussions. The focus group sessions were moderated by a trained Education Department faculty member. A script was developed for the moderator to guide the discussion. The moderator used the script that explained the purpose of the focus group, went over the focus group rules, and reinforced the confidentiality of all the information shared.

Although there are no general rules as to the optimal number of focus group discussions, researchers state that four focus groups are generally sufficient, but that consideration of response saturation should be made after the third focus group discussion (Nyamathi & Shuler, 1990). Guest et al. (2017) stated that within two to three more than 80%, and within three to six focus groups 90% of all themes were discoverable. Three focus groups were also enough to identify the most prevalent themes within the data set. The number of focus groups in this study was guided by theme saturation. After conducting five focus group sessions, it has been observed that the information collected was becoming repetitive, as no new themes were emerging. Therefore, it was decided that data saturation has been reached.

### Data analysis

Content and thematic analysis methodologies were used to analyze the data collected in this study. Content analysis refers to the process in which presentations of behavior or qualitative data from self-reports are analyzed (Karataş, 2015). Content analysis is more related to the initial analysis and the coding process, where researchers look for redundant and similar codes (Humble & Mozelius, 2022). The thematic analysis occurs after the coding process as researchers aggregate similar codes to form major concepts or themes. Basically, thematic analysis converts qualitative data into quantitative data. Once data is transcribed, it is reviewed repeatedly so that the researcher can identify trends in the meaning conveyed by language. The themes identified are re-analyzed so that they become more refined and relevant and given codes (Boyatzis, 1998; Braun & Clarke, 2006). The researcher can then annotate the transcript with the codes that have been identified. In this study, we started with the content analysis as a more basic way of approaching the data material, and then proceeded with the thematic analysis to detect, analyze, and report themes, as well as organize and describe data in dimensions. As distinct and fundamental qualitative approaches, the two should be used by qualitative researchers as transparent structures provide researchers with clear and user-friendly methods for analyzing data (Vaismoradi et al, 2013).

Each student was assigned a code to safeguard their anonymity and confidentiality during the study. The name of the institution was also taken out of the transcribed focus group sessions. The researcher ran a pilot focus group with some students who were not in the sample to verify the understandability of the questions for the focus group interviews’ reliability and validity.

When there were any discrepancies, the meeting platform’s transcriptions were compared to the audio recordings and modified. The written data was afterwards uploaded to the MAXQDA 2020 program, which allows for systematic data analysis (Kuckartz & Rädiker, 2019). The earliest codes were constructed using an inductive approach, and codes that were connected to each other were grouped together and assigned names. Following that, the emerging themes were explained in a way that readers could comprehend. Finally, the researcher provided an interpretation of the findings as well as supporting images.

## Results

### Research question 1

**Fig. 1:**
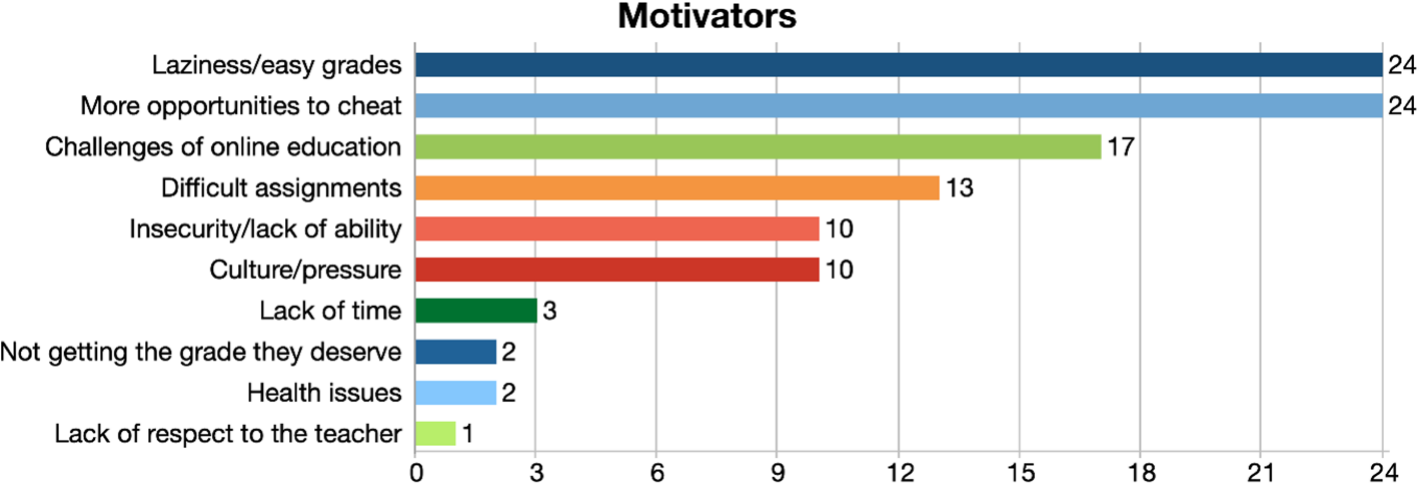
Motivators for contract cheating

The first question analyzed students’ perceptions regarding why more students got engaged in contract cheating during the pandemic. In line with the statements of the participants, the motivators category was defined with ten different codes in order of frequency: wanting to get easy grades, having more opportunities to cheat, challenges of online education and difficult assignments, culture/pressure, and insecurity/lack of ability emerged as some major motivators (Fig. 1).

The participants’ statements regarding the major motivators are below:

“For starters, the online education sort of opened the window of opportunity for students. Now when you see a before and after image, you would think that before we didn’t have access to these sorts of resources. People usually were very busy going to day to day classes. They didn’t have time to research these sort of things. I believe that because people had a bit more free time to do many activities or do whatever during the pandemic because of online education or online learning, they were able to come across these resources through search engines and were able to practice how to use these facilities.” (FG3-1).

Among the motivators, the challenges of online education and the difficulty of assignments in online learning were also mentioned as a reason for resorting to contract cheating. Participants mentioned that students had difficulty accessing information and could not focus during online education:

“Although there are office hours, maybe they need face to face with the professor, in order to learn how to write it properly, because personally when I had an essay writing class, it was easy for me because when we wrote one paragraph, we would review it one on one with the professor. So I feel like that’s why when it came to online, the percentage got a lot higher than when we were on campus.” (FG5-3).

An interesting code that came out of student responses was the culture in Kuwait. Participants mentioned that Kuwaiti children grow up in an environment where everything is done for them. Another element of the culture is the pressure on students to get good grades and graduate with a high GPA to be eligible for government jobs, therefore cheating is considered almost acceptable.

“In Kuwait, in particular, culturally speaking, because of how Kuwaitis were raised or brought up or how they live through an environment of luxury and lack of hardship, to say the very least, it led them to this mindset where they could do these things because they have the option of doing so because it’s so easy to them.” (FG3-1).

### Research question 2

**Fig. 2:**
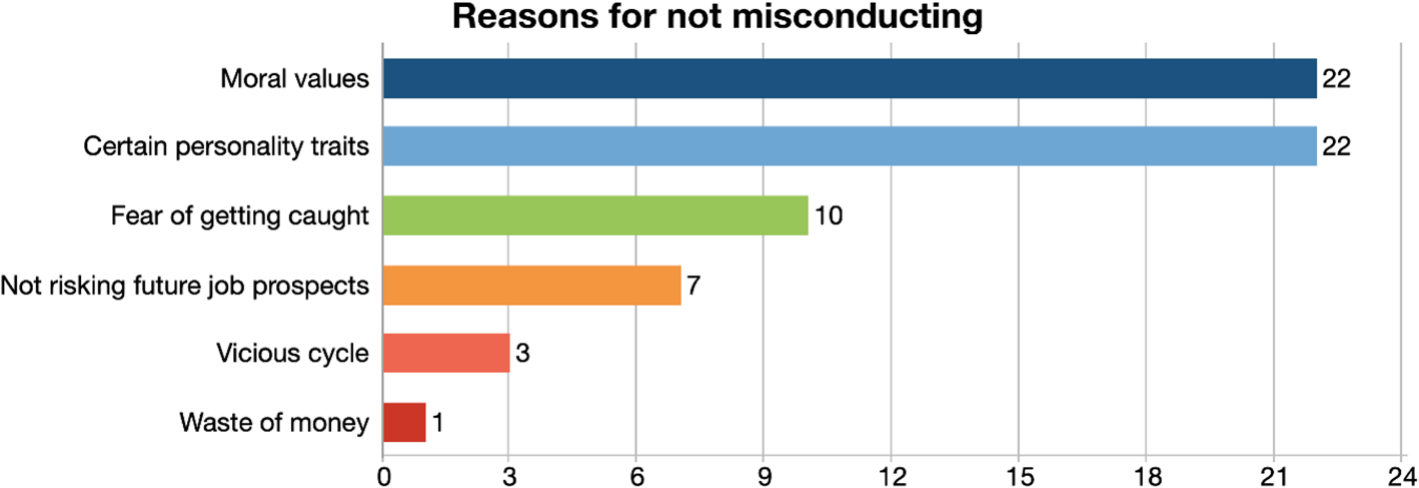
Reasons for not misconducting

When participants were asked what stopped some students from contract cheating during the online education, their responses revealed six different codes. The major deterrents of cheating emerged as moral and religious values and having certain personality traits. Some minor deterrents were fear of getting caught, not wanting to risk future job prospects, and getting trapped in a vicious cycle, also seeing contract cheating as a waste of money (Fig. 2).

One of the most popular responses was the student’s moral values as a reason for not cheating in their assignments.

“Some students, have strong moral values. So, no matter what challenges they face, they would not resort to cheating, because they believe that it’s wrong.” (FG1-2).

Kuwait is officially an Islamic country and Kuwaitis are quite religious people. This was also perceived as part of the non-cheating students’ set of moral values.

“Religion definitely does play a role because in Islam we know that if you cheat to get yourself success, everything you earn from that success is going to be forbidden upon you, so you won’t benefit in the end. But nowadays the religious commitment is not that big.” (FG4-2).

**Fig. 3:**
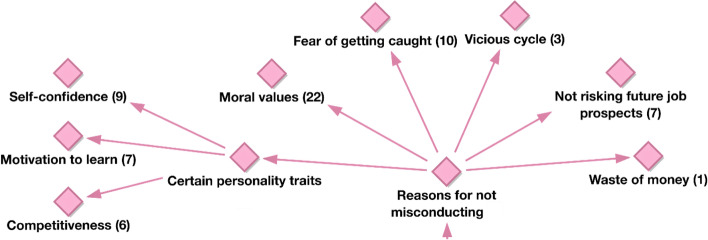
Hierarchical code—subcodes model of reasons for not misconducting

Another code that the participants’ responses revealed was certain personality traits. Under this code participants mentioned three different subcodes: self-confidence, motivation to learn, and competitiveness (Fig. 3).

Participants mentioned that when students have enough self-confidence, motivation, and competitiveness, they do not need any help, they are excited about their achievements, and they cannot trust anyone else to do their work for them.

“I think some students do not like to depend on other people to do their work. Also they are not trustworthy like they can’t trust them to do their work as they feel more confident doing their own work. In this way they will improve themselves to become a better person.” (FG2-4).

“Eventually people we will come back to fully on a real life and we’ll be in a position where you cannot cheat. So, in order to enhance our knowledge, or to do better in future classes, they wouldn’t cheat, so they can actually learn something.” (FG5-4).

Another reason why some students did not cheat was the fear of getting caught, according to students’ perceptions. Participants mentioned that some students did not resort to cheating because they were afraid of the outcomes in case they get caught. This was also similar to another deterrent mentioned by students as not wanting to risk future job prospects. Some quotes below exemplify such perceptions:

“I think one of the biggest things that most students fear when it comes down to plagiarizing or cheating is getting caught. But I also think what would devastate a student is if the teacher or the instructor make an example out of the student. Because if you pull out their assignment in front of the entire class and say that ‘this is plagiarized, and because of that, I will give you a zero’, you know you would be set as an example, and I think that would break a student, and so I think that thought or the fact that you’re getting caught. And then being exposed is what really scares or that fear that set a lot of students aside from wanting to plagiarize or cheat.” (FG1-2).

“This would affect them in long term, and they wouldn’t be able to do stuff that normal person would be able to do and complete their assignments and all of that stuff. They will have problems in their jobs later on their lives.” (FG4-3).

“I think it’s all a certain mindset. Some students don’t cheat because they realize there’s no meaning to it in a sense that if you do cheat your whole life, you’ll keep cheating… if you cheat now, you’re going to cheat in your whole life and there’s no point to it.” (FG3-1).

### Research question 3

**Fig. 4:**
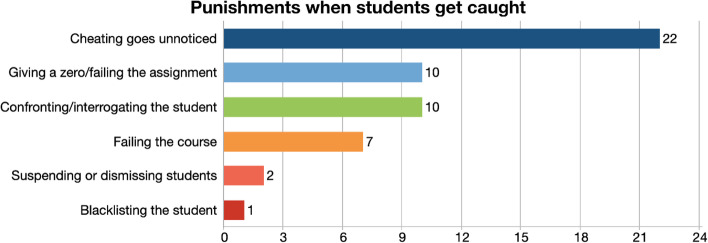
Punishments when students get caught

Students were asked about their perceptions towards the consequences cheating students faced. Did cheaters get caught? Did they get penalized sufficiently when they were caught? Did cheating ever go unnoticed? The responses revealed six different codes, which could be summarized as most of the time cheating went unnoticed, and when it was noticed, they received certain punishments ranging from failing the assignment and/or the course or being suspended from the university (Fig. 4).

The most striking response as analysis revealed was most participants thought instructors did not even realize that cheating took place.

“I think it’s less likely that the professor will catch the student until and unless they know the student and his past assignments. Because the professors don’t know anything about them. They don’t know how they’ve been doing. I think they get their assistants to check the paper so they are very less likely they can catch the culprit.” (FG4-3).

In case students got caught contract cheating, the most common consequence they faced was getting a zero and failing that specific assignment. Failing the entire course or being suspended from the university for a semester and blacklisted on a list that circulates among faculty members were also mentioned by some participants was another repercussion mentioned by some participants.

“I had no experience with people getting caught with cheating, but like usually if they get caught they just get a zero for the essay. For that particular assignment, not for the whole course.” (FG4-1).

Students also talked about being interrogated by the instructor as a consequence of cheating. This interrogation sometimes took place in private, but sometimes in public, in front of their peers, which was a big source of embarrassment for the student:

“To make it even better, they should do the punishments publicly so other students can see this person is being punished for this reason so they can scare everyone else from being humiliated in front of the class. Well, what we did in our old school if someone cheats, they rip the paper on the spot and then kick the student out the class.” (FG5-1).

### Research question 4

**Fig. 5:**
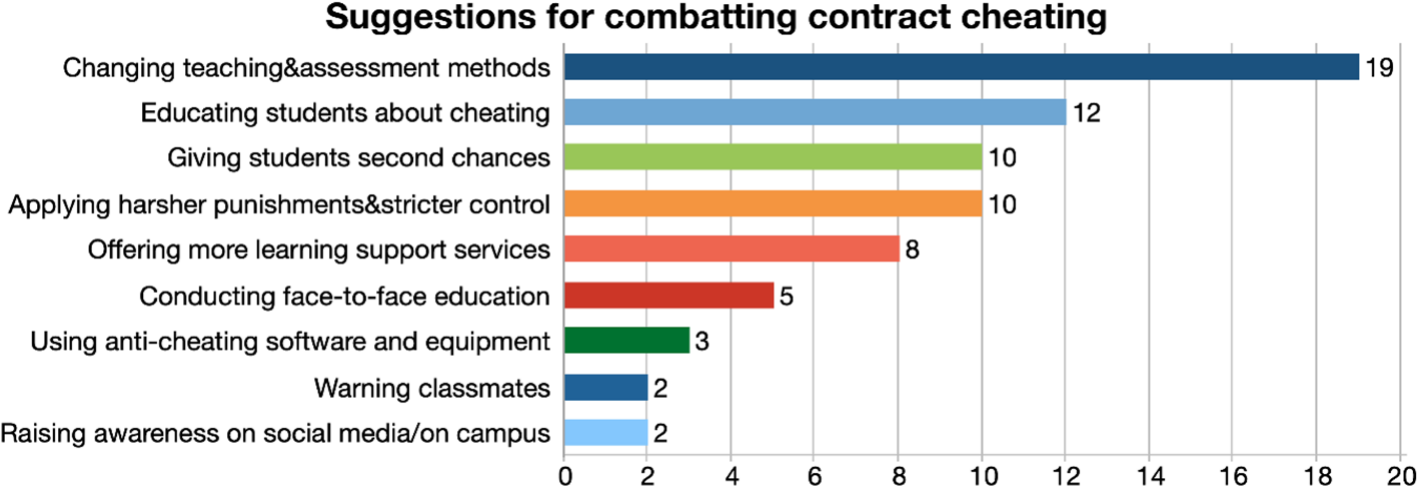
Suggestions for combatting contract cheating

The final research question of the study focused on solutions to this academic misconduct and asked students to make suggestions to prevent this problem. Students were reminded to consider all the stakeholders in their suggestions, such as students, instructors, and the administration of the institution in which they are studying. Their responses revealed nine codes as seen in Fig. 5. The solutions could be classified as positive and negative ones, with the positive, more nurturing solutions being changing teaching and assessment methods, educating students about cheating, giving students second chances, offering more learning support services, conducting face-to-face education and raising awareness on social media and on campus. Some students were in favor of more punitive solutions, such as applying harsher punishments and stricter control, and using anti-cheating software and equipment.

The suggestion with the highest frequency was changing the teaching and assessment methods. Participants mentioned that education system relies too much on rote learning, memorization and should include more hands-on assignments and projects. As a result, assessment types should move from multiple choice, written exams to more applied methods and performance assessment.

“That’s the only way I could see this work is if the entire like education industry changed the way they move forward with their teaching and their learning, make it more practical with experience… more than just about grades. The fact that you know your grades are on the line and students compare one of their grades, their GPA over the other, the pressure is intense, and that’s where you know people resort to things that are much easier. But if it’s more about, having fun, learning, experiencing, things are quite practical to the world out there specifically tailored to what they want to do in the future, that would completely eliminate the. You know, the problem of plagiarism and cheating or whatnot, because then they’re doing something they love doing.” (FG1-2).

The next common code was educating students about cheating. Participants mentioned that students need to be educated about cheating and given clear warnings about the outcomes. Some participants expressed the need for a more nurturing environment for students and that they should be given a second chance when caught. Offering more learning support services was also proposed as a solution as some participants to encourage students to seek more help from legal sources.

“I do believe that a severe punishment would refrain the students from cheating, but what would be an even better learning objective to make them understand that cheating would be wrong, plagiarizing would be wrong is to let them understand how severe it is beforehand so they wouldn’t do it in the first place, not by punishing them if they cheated. Making it sink down deep end that this is how severe cheating is… this is what will happen. Because some students don’t really understand the full gravity of what they’re doing and what would happen if they’re caught.” (FG3-1).

“Maybe they’re lazy, but in most cases they need help, they need people to help them with things they don’t understand. They need help with things that they probably don’t know. Or maybe they weren’t focused in class on a particular day. Students need help, like education is not easy. It’s a learning process. We as people learn through mistakes and experiences, but we also need a guiding hand in order for us to succeed in life and for us we should not focus solely on punishments because at the end of the day students or these young people are the future of the nation of Kuwait of this country. If we can guide them to a better path instead of punishing them and then going on a very darker or very negligent path, it would have been much better. Not only for us,

Other codes that came out of our analysis were conducting face-to-face education, using anti-cheating software and equipment in exams to detect cheating, warning classmates about the outcomes of cheating, and raising awareness on social media. Some participants asked for harsher punishments to combat cheating.

## Discussion

The first research question on the motivators of contract cheating revealed the fact that some students outsourced their tasks because they just wanted to get easy grades and online learning made this a possibility by providing more opportunities to cheat. Many students were just looking to get by and pass the course because the shift to online education has drastically affected their ability to learn and retain information, and they only intended to cheat in the short-term.

According to Gallant (cited in Dey, 2021), there was probably increased cheating because there were more temptations and opportunities. When colleges shut down or restricted in-person access, students were taking exams in their bedrooms, with unrestricted access to their phones and other information technologies. This spurred cheating to take on new and different forms. Regarding students cheating in online courses, if students feel anonymous and unlikely to be adequately monitored, they may assume that the likelihood of being caught cheating is virtually zero and cheat more in online classes using online resources. Previous research has shown that participants had a higher propensity to cheat when chances of being caught were less likely (Kajackaite & Gneezy, 2017). Despite being supervised through a web camera, the teachers cannot control the surroundings or the computer screens of the students. In class, students are regularly monitored and watched and thus are less free to consult sources of information, but with the physical distance, those odds decrease, and so cheating increases.

College students could not help but want to be a part of the herd since they did not want to be left out when their peers were earning good scores without putting in any effort. This is further reinforced by research findings that students are less inclined to cheat when they believe their peers are trustworthy and the misconception of “everyone else is doing it” encourages cheating (Carpenter et al., 2006; Daniels et al., 2021; Turner & Uludag, 2013). Observing their peers’ cheating activities in online classes through group chats, as our participants’ responses reveal, encourages more students to cheat, especially after the initial shock has worn off and they felt more at ease, in the second semester of online education.

The difficulties of online education have been cited in various research as a factor that contributed to contract cheating. Along with the opportunities online learning provided, stress and pressure started building up and the pandemic essentially intensified a feeling of potential loss among college students. Asking questions during exams was difficult without the in-person experience. Students were able to ask questions via email or attend virtual office hours, but many missed the ease of raising a hand and getting a question answered in real time. According to a study conducted in Vietnam (Tran, et al., 2021), students had generally negative feelings toward online education, with 63.31% of respondents stating that they disliked online exams and 64.8 percent stating that online learning was only marginally effective and only a temporary solution. The difficulties in assessing and testing online, as well as not understanding the course and communicating with peers, were identified as negatives.

With the outburst of the pandemic, many students found their surroundings transformed completely. Such a change probably caused an increased fear of loss with students being away from their friends and normal social environment, away from the usual learning atmosphere and resources they are used to. They developed a fear of losing social connections, falling behind in class, losing internship and career opportunities, etc. In a study that surveyed students from all over the USA (Hoyt et al., 2021), students reported that the loss of their social life had a major influence on their mental state during the pandemic. When these results are considered with the established idea that increased fear of loss can cause a biological reaction that increases dishonest behavior, it can reasonably be assumed that one of the primary reasons colleges all over the world detected abnormally high cheating rates among their students is an increase in a fear of loss (Arie, 2021). Similar findings were seen in other research, including as in Hong Kong, where students said they struggled to maintain self-discipline when studying alone on online platforms (Mok et al, 2021); students experienced stress, worry, and pressure as a result of the pandemic (Sahu, 2020), and they did not find online learning to be totally rewarding, particularly when they experienced disruptions during online classes due to insufficient educational and institutional assistance (Fauzi & Sastra Khusuma, 2020; Xie & Yang, 2020).

The purpose of our second study question was to examine students’ perspectives of the motivations for not cheating. Students’ responses emphasize the importance of students’ own moral compass, as well as particular personality traits like self-confidence, ambition to learn, and competition, as key deterrents to cheating. This is consistent with research that emphasizes the role of attitudes and beliefs in preventing academic misconduct and promoting an ethical culture (Rundle et al., 2019; Grym and Liljander, 2016) Strong individual views and ideals regarding integrity, according to Reedy et al. (2021), minimize the likelihood of students cheating. Following these two major deterrents, fear of being caught emerges as a third code, which is corroborated by the findings of an Australian study by Rundle et al. (2019). Three significant predictors of fear of detection and punishment were identified in Rundle’s regression analysis (Machiavellianism, narcissism, and consistency of interest), implying that students who scored high on these are more likely to report fear of detection and punishment as a reason for not engaging in contract cheating. These findings imply that appealing to students’ values and beliefs while conveying clear messages about academic integrity could be an effective method for improving the integrity of online and offline exams.

The study’s third finding concerned the implications of cheating. Students were asked what the consequences and punishments were when cheaters were caught. Surprisingly, the highest frequency was observed in the code that cheaters were not generally caught, and contract cheating went unnoticed. This finding is intriguing in a way as we are not sure whether instructors really fail to recognize cheating or tend to ignore it and not take any action as it is difficult to present hard evidence to prove contract cheating. Research found that faculty were able to identify 62% of contract cheating when they were advised to specifically look for it (Dawson & Sutherland-Smith, 2019); but when they were unaware of the possible presence of contract cheating, they could not detect any (Lines, 2016). Although Erguvan’s study (2021) on faculty awareness of contract cheating found that faculty members are confident in their ability to spot it even when cases are detected, teaching staff are concerned that proving cheating may be difficult (Walker & Townley, 2012). Faculty members frequently express concerns about cheating during online education, but they have not always been able to detect and punish cheating as they would like to, due to a lack of security measures, reliable plagiarism detection tools, and training on online assessment and cheating prevention measures (Meccawy et al, 2021). Because of the problem’s complexity and the difficulties to solving it, faculty members may simply choose to ignore it (Coren, 2011; McCabe, 2005).

When faculty members detect students cheating on an assignment, the most typical repercussions include failing the work and being interrogated by the teacher, sometimes in private and sometimes in public. Students, on the other hand, stated that failing an assignment is not a strong enough deterrent to cheating because these assignments are often so little in proportion that they have little impact on students’ total grade in the course. Another study found that academic dishonesty is frequent among Kuwaiti university students because the danger of detection and severity of sanctions for academic misconduct is minimal (Alsuwaileh et al., 2016). Participants believe academic dishonesty remains widespread because sanctions are not enough. According to most of the participants, embarrassment is the only informal sanction for academic dishonesty, and they would be embarrassed more by the lecturer than by their friends or families.

According to the findings of the fourth research question, students believe that the existing educational system merely promotes students to memorize and does not teach them the real skills they need in their jobs. Students expressed their desire for a change in the university's educational and assessment techniques. Indeed, there is a growing body of research on the function of evaluation in contract cheating prevention and detection. Some suggest increasing the use of invigilated (Clarke & Lancaster, 2006; Lines, 2016) and in-class viva voce examinations (Carroll and Appleton, 2001) to reduce the potential for cheating. Others focus on reducing motivations to cheat through increasing student engagement by choosing personalized topics (Sutherland-Smith, 2013), and using authentic assessment, which aims to engage students in “real-world” tasks (Collins et al, 2007; QAA, 2020).

However, some researchers are skeptical about the impact of changing the assessment in curbing contract cheating and suggest that authentic assessment does not necessarily assure academic integrity and that educators need to be aware that cheating may take place even in applied and “authentic” exams such as oral exam/viva or practical exam (Harper et al., 2021; Ellis et al, 2020).

Text-rich forms of assessment, according to Harper et al. (2021), should keep their place in university assessment strategies, not because they are impervious to contract cheating, but because faculty get more proficient in detecting cheating in written assignments like research papers, which enable them to develop more personalized relations with students. This supports the students’ belief that, in addition to assessment, a shift in pedagogy could play a role in minimizing contract cheating.

Our research suggests that students want to be educated about academic integrity and given explicit warnings about the consequences, but they also believe punishments should be severe enough to work as deterrents. The rational choice theory may offer hints about how to curb the plagiarism problem in this regard. Universities should increase the benefits associated with non-plagiarized papers and publicly circulate information about plagiarism; otherwise, any punishments or sanctions will not be deterrent to plagiarism. Academic integrity values may be fostered, and students can become familiar with this culture through course objectives and activities. A system of progressive educational punishment might likewise be implemented (Cinali, 2016; Mervis, 2012). Faculty members and university administrators should diminish the benefits of plagiarism and increase the costs and the probability of detection. If students still choose to plagiarize, they must take higher risks into account; otherwise, they need to either be experts in hiding plagiarism or make greater effort in producing plagiarism that is hard to detect, which will reduce the time-saving benefits of plagiarism (Collins et al., 2007) and in turn reduce the number of students committing plagiarism.

## Conclusion and recommendations

The findings of this study reveal that as per students’ perceptions, online learning has driven more students to contract cheat, primarily by approaching an essay mill or a tutor and paying them to do the work for them. Students expressed they were tempted by the opportunities presented by online learning, such as not having any proctoring or an obligation to turn on their webcams during exams, ease of finding a tutor to do the work at a very affordable rate, and not having the motivation and skills to cope with the challenges of online classes, therefore choosing the easy way. Some students described the feeling as “being part of the herd,” which could be compared to the new trendy acronym FOMO (fear of missing out), that basically refers to the feeling or perception that others are having more fun, living better lives, or experiencing better things than you are, which is often exacerbated by social media.

To summarize, academic integrity violations have been on the rise as a result of COVID-19-mandated online or hybrid education systems which may tempt many students to continue using their tried and tested methods of cheating when they return to face-to-face instruction. Therefore, violations of academic integrity necessitate a rethinking of teaching and evaluation methodologies. Higher education institutions must adapt to the changing contract cheating marketplace and ensure that the faculty are aware of contract cheating and can recognize the indicators of contract cheating. Students should be given the message that their tutors are aware of contract cheating services. To keep up with the constant changes in technology, academic integrity processes must be current, resilient, and assessed on a regular basis.

If we do not take immediate action, contract cheating will likely reach epidemic proportions. We need to take a comprehensive approach that includes a focus on assessment design, a strengthened culture of integrity, and robust technical tools. We should also urge academics to perform ongoing research on ways to improve academic integrity during and post pandemic higher education instruction.

## Limitations

The author is confident this paper will add significant value to the body of existing literature; however, we cannot be sure that the focus groups have captured a representative sample of students studying in higher education institutes in Kuwait. It is also important to note that the study is limited to the experiences and assumptions of students who participated in the study and therefore the findings should not be generalized.

## Data Availability

The qualitative data that support the findings of this study are available on request from the corresponding author, DE. The data are not publicly available as they contain information that could compromise the privacy of research participants.

## Abbreviations

COVID-19: Coronavirus disease
MAXQDA: Max Qualitative Data Analysis
